# Low Ki67/high ATM protein expression in malignant tumors predicts favorable prognosis in a retrospective study of early stage hormone receptor positive breast cancer

**DOI:** 10.18632/oncotarget.12622

**Published:** 2016-10-12

**Authors:** Xiaolan Feng, Haocheng Li, Elizabeth N. Kornaga, Michelle Dean, Susan P. Lees-Miller, Karl Riabowol, Anthony M. Magliocco, Don Morris, Peter H. Watson, Emeka K. Enwere, Gwyn Bebb, Alexander Paterson

**Affiliations:** ^1^ Department of Oncology, BC Cancer Agency-Vancouver Island Center, Victoria, British Columbia, Canada; ^2^ Faculty of Medicine, The University of British Columbia, Vancouver, British Columbia, Canada; ^3^ Department of Oncology, Tom Baker Cancer Centre and University of Calgary, Cumming School of Medicine, Calgary, Alberta, Canada; ^4^ Department of Community Health Science, TRW Building, University of Calgary, Calgary, Alberta, Canada; ^5^ Functional Tissue Imaging Unit, Translational Research Laboratory, Tom Baker Cancer Centre, Calgary, Alberta, Canada; ^6^ Translational Research Laboratory, Tom Baker Cancer Centre, Calgary, Alberta, Canada; ^7^ Department of Biochemistry and Molecular Biology, Health Science Building, University of Calgary, Alberta, Canada; ^8^ Department of Anatomic Pathology, H. Lee Moffitt Cancer Center, Tampa, FL, USA; ^9^ Department of Pathology, BC Cancer Agency-Vancouver Island Center, Victoria, British Columbia, Canada

**Keywords:** ATM, Ki67, early stage hormone receptor positive breast cancer, automated quantitative immunofluorescence analysis, disease specific overall survival

## Abstract

**Introduction:**

This study was designed to investigate the combined influence of ATM and Ki67 on clinical outcome in early stage hormone receptor positive breast cancer (ES-HPBC), particularly in patients with smaller tumors (< 4 cm) and fewer than four positive lymph nodes.

**Methods:**

532 formalin-fixed paraffin-embedded specimens of resected primary breast tumors were used to construct a tissue microarray. Samples from 297 patients were suitable for final statistical analysis. We detected ATM and Ki67 proteins using fluorescence and brightfield immunohistochemistry respectively, and quantified their expression with digital image analysis. Data on expression levels were subsequently correlated with clinical outcome.

**Results:**

Remarkably, ATM expression was useful to stratify the low Ki67 group into subgroups with better or poorer prognosis. Specifically, in the low Ki67 subgroup defined as having smaller tumors and no positive nodes, patients with high ATM expression showed better outcome than those with low ATM, with estimated survival rates of 96% and 89% respectively at 15 years follow up (*p* = 0.04). Similarly, low-Ki67 patients with smaller tumors, 1-3 positive nodes and high ATM also had significantly better outcomes than their low ATM counterparts, with estimated survival rates of 88% and 46% respectively (*p* = 0.03) at 15 years follow up. Multivariable analysis indicated that the combination of high ATM and low Ki67 is prognostic of improved survival, independent of tumor size, grade, and lymph node status (*p* = 0.02).

**Conclusions:**

These data suggest that the prognostic value of Ki67 can be improved by analyzing ATM expression in ES-HPBC.

## INTRODUCTION

The incidence of early stage breast cancer (stage I-III) has been increasing over the last 20 years largely due to the introduction of nation-wide mammogram screening program (Canadian Cancer Statistics 2015). At the time of the collection of these tissues (and excluding the 20% of in-situ cases) there would likely have been around 50% of patients with stags I, with around 40% with stage II and III, the remainder being stage IV or unknown. Although it is interesting that we do not have population data readily available for treatments given, approximately 60% of the patients with early stage breast cancer would have been given only adjuvant endocrine therapy.

Early stage hormone receptor-positive breast cancer (ES-HPBC) has a relatively good prognosis; it is, however, molecularly diverse and distinctive for late relapse. Consequently, a certain subset of patients with ES-HPBC has a poor prognosis. ES-HPBC can be broadly divided into luminal A [estrogen receptor (ER)/progesterone receptor (PR) strong positive, human epidermal growth factor receptor 2 (HER2) negative, well differentiated and less proliferative] and luminal B (ER/PR low to moderate positive, poorly differentiated and highly proliferative) subtypes with a 10-year breast cancer-specific overall survival (DSOS) of 92% (95% CI 90-94) and 79% (95% CI 74-85) respectively (*p* < 0.001) [1].

Ki67, a protein expressed exclusively in proliferating cells [2], is commonly used to determine luminal subtypes in ES-HPBC [1], and is adopted as a prognostic marker in breast cancer in general [3, 4]. However, the prognostic value of Ki67 in ES-HPBC is a matter of controversy [5–8]. A wide variety of assay methods and significant inter-observer variability [9–12] have made it difficult to clearly and consistently determine the value of Ki67 as a standalone prognostic marker. Nevertheless, Ki67 is commonly used in clinical decision-making, particularly to select which patients with supposedly good prognosis could avoid chemotherapy. While the weight of evidence is against such use [5–8], one cannot ignore the value of Ki67 as part of a prognostic index. It is notable that Ki67 is among the genes carrying the greatest weight in the recurrence score-calculating algorithm of Oncotype Dx, which is a validated prognostic and predictive assay with extensive use in ES-HPBC [13, 14]. Such use suggests that Ki67 is best used along with other biomarkers, which would improve its prognostic utility while avoiding pitfalls that emerge from variability in assay methods.

We and others previously showed that ataxia telangiectasia mutated (ATM), a protein that is critical in maintaining genomic stability [15], is significantly reduced in breast cancer as compared to normal breast tissue. We also showed that reduced ATM expression is associated with poor clinical outcome in early stage breast cancer [16–18]. Moreover, low ATM is strongly related to known poor prognostic factors such as larger tumor size, positive lymph nodes (LN), and high grade in ES-HPBC [18]. ATM is, however, not an independent prognostic factor in a multivariable analysis in ES-HPBC [18].

In this study, we used immunohistochemistry (IHC) and automated semi-quantitative digital analysis to detect and quantify ATM and Ki67 in resected primary tumors from patients with ES-HPBC. We hypothesized that the combination of both biomarkers would be a better prognostic indicator than either biomarker alone. Specifically, we assessed the breast cancer specific survival as an outcome using both biomarkers in LN negative or positive (LN = 1-3) ES-HPBC. We further asked if ATM status was useful to stratify the outcome of ES-HPBC patients, particularly in the subgroup with low Ki67 expression.

## RESULTS

### Patient characteristics

Our study adhered to the REMARK criteria for the study of biomarkers [19]. The clinical and tumor characteristics of the patients in the initial clinical cohort (*n* = 819), the selected TMA cohort (*n* = 532) and the final cohort (*n* = 297) are shown in Appendix 1B. It is notable that the patients’ characteristics and clinico-pathological features including age, tumor size, grade, LVI and LN in these three cohorts are similar (insignificant p value in Appendix 1B), suggesting that the final cohort could be representative of our initial clinical cohort. For the final cohort, we only included ES-HPBC (stage I-III) patients with Ki67/ATM data and confirmed ER/PR positivity and HER2 negativity (Appendix 1A). There were thus differences between the final cohort and the other two cohorts (the clinical and TMA cohorts) with regard to stage, ER/PR, and HER2 status (Appendix 1B).

The clinical and tumor characteristics of these patients in the final cohort, which was subdivided into four groups based on ATM and Ki67 expression levels, is shown in Table 1. Briefly, mean age at diagnosis was 65 years. The majority of cases had smaller tumors (96%; T1/T2), and were non-high grade (80%; grades 1/2), LN negative (74%) and stages I and II (93%), indicative of the early staging of this cohort. Sixty percent of patients had adjuvant radiation treatment (RT). All patients (100%) received adjuvant endocrine therapy in the form of tamoxifen and 19% of patients also received aromatase inhibitors as adjuvant endocrine treatment. No patients in this cohort received adjuvant chemotherapy.

**Table 1:** Comparison of patients’ characteristics and clinico-pathological features of breast tumors in high and low ATM/ Ki67 groups in the final cohort

Baseline Characteristics	Full Cohort *N*=297 (100%)	Low Ki67 Group		High Ki67 Group		Low Ki67 Group
		High ATM /Low Ki67 *N*=145 (49%)	Low ATM/ Low Ki67 *N*=105 (35%)	High ATM/ High Ki67 n=21 (7%)	Low ATM/ High Ki67 N=26 (9%)	Low Ki67/high ATM vs Low ATM/ Low Ki67 p value
Age median (min-max)	65 (38-96)	63(38-96)	68(38-89)	68(43-78)	68(50-86)	0.150
						
Tumor size						
T1/T2 (<5cm)	285 (96%)	141 (48%)	99 (33%)	21 (7%)	24 (8%)	0.329
T3/T4 (≥5cm)	12 (4%)	4 (1%)	6 (2%)	0 (0%)	2 (1%)	
Missing	0 (0%)	0 (0%)	0 (0%)	0 (0%)	0 (0%)	
						
Grade						
1	77(26%)	48 (16%)	26 (9%)	1 (0.5%)	2 (1%)	0.090
2	161 (54%)	80 (27%)	61 (20%)	10 (3%)	10 (3%)	
3	47 (16%)	10 (4%)	15 (5%)	9 (3%)	13 (4%)	
Missing	12 (4%)	7 (2%)	3 (1%)	1 (0.5%)	1 (0.5%)	
						
LVI						
Negative	180 (61%)	90 (30%)	64 (21%)	12 (4%)	14 (5%)	0.584
Positive	50 (17%)	20 (7%)	18 (6%)	4 (1%)	8 (3%)	
Missing	67 (22%)	35 (12%)	23 (8%)	5 (2%)	4 (1%)	
						
LN						
0	220 (74%)	114 (38%)	82 (28%)	13 (4%)	11 (4%)	1.0
>0	77 (26%)	31 (10%)	23 (7%)	8 (3%)	15 (5%)	
Missing	0 (0%)	0 (0%)	0 (0%)	0 (0%)	0 (0%)	
						
Stage						
I	168 (57%)	94 (32%)	63 (21%)	7 (2%)	4 (1%)	0.012
II	108 (36%)	47 (16%)	29 (10%)	12 (4%)	20 (7%)	
III	21 (7%)	4 (1%)	13 (4%)	2 (1%)	2 (1%)	
Missing	0 (0%)	0 (0%)	0 (0%)	0 (0%)	0 (0%)	
						
ER/PR status						
Positive	297 (100%)	145 (49%)	105 (35%)	21 (7%)	26 (9%)	
Negative	0 (0%)	0 (0%)	0 (0%)	0 (0%)	0 (0%)	
Missing	0 (0%)	0 (0%)	0 (0%)	0 (0%)	0 (0%)	
						
HER2 Status						
Positive	0 (0%)	0 (0%)	0 (0%)	0 (0%)	0 (0%)	
Negative	297 (100%)	145 (49%)	105 (35%)	21 (7%)	26 (9%)	
Missing	0 (0%)	0 (0%)	0 (0%)	0 (0%)	0 (0%)	
						
RT						
Yes	178 (60%)	97 (33%)	57 (19%)	9 (3%)	15 (5%)	0.107
No	113 (38%)	47 (16%)	44 (15%)	11 (4%)	11 (4%)	
unknown	6 (2%)	1 (0.5%)	4 (1%)	1 (0.5%)	0 (0%)	
						
Chemo						
Yes	0 (0%)	0 (0%)	0 (0%)	0 (0%)	0 (0%)	
No	297 (100%)	145 (49%)	105 (35%)	21 (7%)	26 (9%)	
						
Endocrine						
Tamoxifen	297 (100%)	145 (49%)	105 (35%)	21 (7%)	26 (9%)	
Aromatase inhibitor	56 (19%)	26 (9%)	18 (6%)	6 (2%)	6 (2%)	1.0

### ATM and Ki67 expression analysis

**Figure 1:**
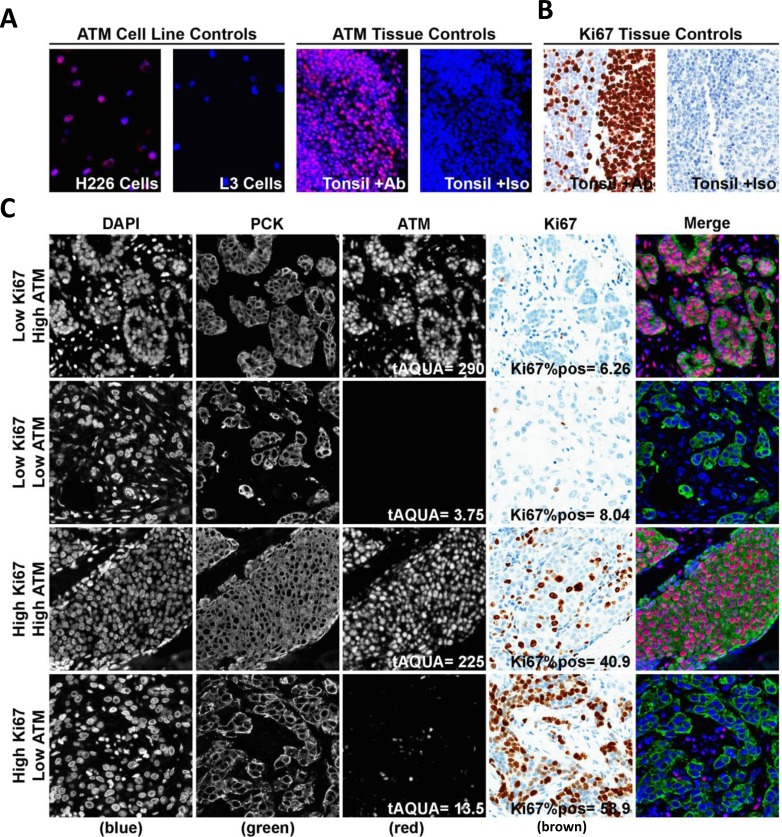

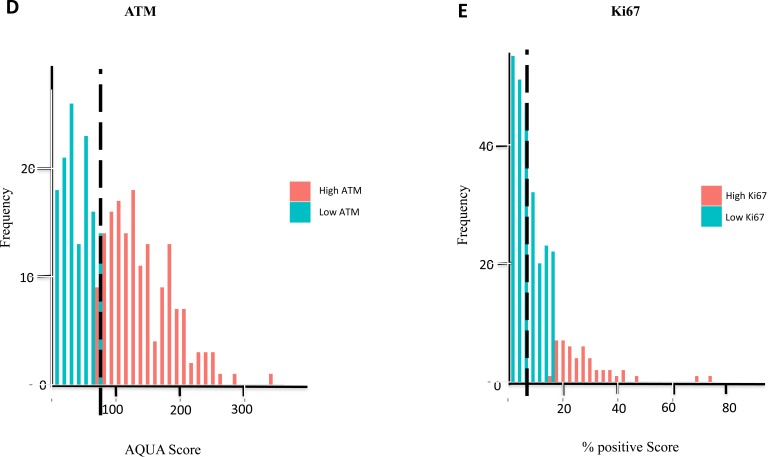
Semi-quantitative fluorescence immunohistochemistry and digital image analysis for ATM and Ki67 in ES-HPBC **A.** Positive and negative controls for ATM were ATM-expressing H226 cells and ATM-deficient L3 cells, as well as in breast cancer tissue with either rabbit IgG or primary anti-ATM antibody. **B.** Normal tonsil tissue stained with the Ki67 or isotype control antibodies served as positive and negative controls respectively for Ki67. **C.** Representative fluorescence images for low Ki67/high ATM, low Ki67/low ATM, high Ki67/high ATM and high Ki67/low ATM expression in ES-HPBC cohort. DAPI-stained nuclei are depicted in blue (first column), pan-cytokeratin-stained malignant cells are depicted in green (second column), and ATM is depicted in red (third column). Haematoxylin-stained nuclei are depicted in blue, while DAB-stained Ki67-positive nuclei are depicted in brown (fourth column). **D.**, **E.** Histograms representing the distribution of ATM expression (D) and Ki67 expression (E) in ES-HPBC. The hashed black line represents median Ki67/ATM expression in this cohort.

ATM protein was detected by fluorescence IHC, and quantified by digital image analysis using AQUAnalysis software [20] (Figure 1). Antibody specificity was confirmed using the L3 ATM-deficient human squamous lung carcinoma cell line and the age-matched H226 ATM-expressing human squamous lung carcinoma cell line as controls (Figure 1A, left two panels) [18]. As expected, no IHC signal was detected in the L3 cells, whereas the H226 showed clear nuclear staining. Positive and negative (by omission of the primary antibody) tonsil tissue controls were included in each run (Figure 1A, right two panels). Normal tonsil tissue stained with the Ki67 or isotype control antibodies served as positive and negative controls respectively for Ki67 (Figure 1B). Representative images of the low Ki67/high ATM, low Ki67/low ATM, high Ki67/high ATM, and high Ki67/low ATM specimens are shown in Figure 1C. ATM expression was determined within each patient tissue core as the average Cy3 pixel intensity within the pan-cytokeratin positive malignant cell area, and represented as an AQUA score (Figure 1C). For each patient sample, the average AQUA score over triplicate cores was used to define the ATM expression score. Ki67 expression was indicated as the percentage of the analyzed tumor area that contained positive (DAB) staining (Figure 1C). The histograms in Figure 1D and 1E indicate the distributions of Ki67 and ATM expression scores. The median ATM AQUA score and Ki67 percent positive score in this cohort were 85.8 (95% CI: 72.3-98.3) and 7.0 (95% CI: 6.2-8.4) respectively (Figure 1D and 1E, hashed black line).

### Association between Ki67/ATM protein expression and clinical outcome

Patients were stratified into subgroups based on high and low Ki67/ATM scores, using independent cut-points identified by X-Tile software [21]. The cut-point was set at an AQUA score of 72.2 for ATM and a percent positive score of 16.9 for Ki67 respectively. As both the high Ki67 groups (high Ki67/high ATM and high Ki67/low ATM) have low study numbers (*n* = 21 and 26 respectively), we focused our further statistical analysis on low Ki67 groups. Patients with low Ki67/low ATM were significantly more likely than their low Ki67/high ATM counterparts to have stage III disease (*p* = 0.012) (Table 1). There were no associations between low Ki67/low ATM and other well-known prognostic factors including tumor size (*p* = 0.177), LN status (*p* = 1.0), or grade (*p* = 0.086) (Table 1), implying that low Ki67/low ATM may constitute an independent prognostic factor.

**Figure 2:**
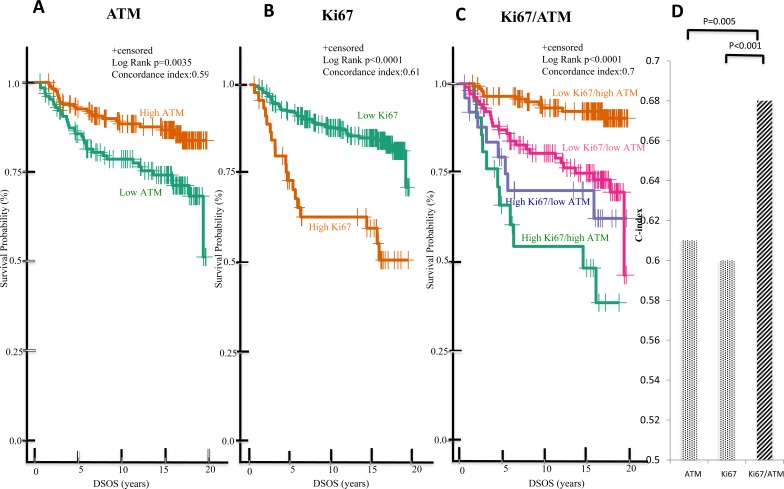
Low Ki67/high ATM expression in malignant tumors predicts favorable DSOS in ES-HPBC (stage I-III) Kaplan-Meier analysis of DSOS between patients with high and low ATM expression alone **A.**, high and low Ki67 expression alone **B.**, and of patients in these four groups (low Ki67/high ATM, low Ki67/low ATM, high Ki67/high ATM, high Ki67/low ATM expression) **C.** C-index was used to evaluate the model prediction accuracy of single (ATM or Ki67) *versus* combined biomarkers (Ki67/ATM) **D.**

**Figure 3:**
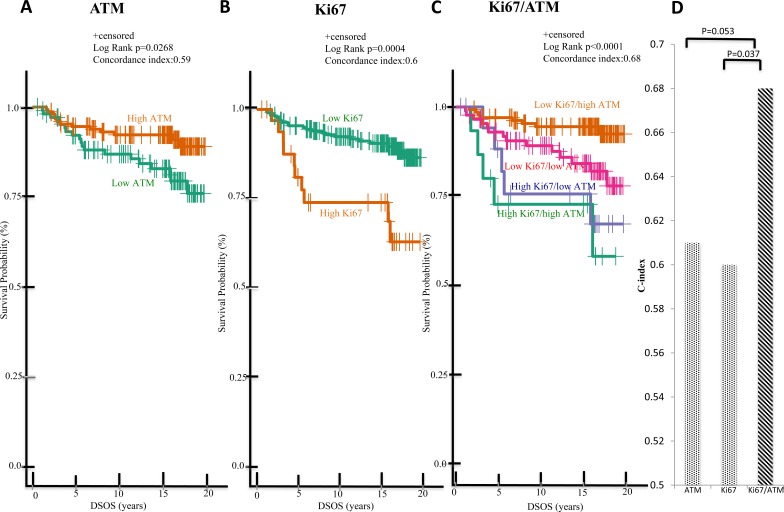
Low Ki67/high ATM expression in malignant tumors predicts favorable DSOS in ES-HPBC (size < 4cm, LN < 4) A subgroup of patients with tumor size < 4cm and LN < 4 were selected for further analysis. Kaplan-Meier analysis of DSOS between patients with high and low ATM expression alone **A.**, high and low Ki67 expression alone **B.**, and of patients in these four groups (low Ki67/high ATM, low Ki67/low ATM, high Ki67/high ATM, high Ki67/low ATM expression) **C.** C-index was used to evaluate the model prediction accuracy of single (ATM or Ki67) *versus* combined biomarkers (Ki67/ATM) **D.**

To further investigate the prognostic value of these biomarkers, patients were separated into sub-groups based on high or low ATM or Ki67 expression, and the survival data were subjected to further analysis using the Kaplan-Meier method. Consistent with previous studies [3, 4, 18], DSOS significantly differed based on ATM or Ki67 alone. For patients with stage I-III HPBC, the estimated 15-year DSOS rates were: 87% and 74% for high and low ATM group respectively (*p* = 0.0035); and 85% and 60% for low and high Ki67 group respectively (*p* < 0.001) (Figure 2A and 2B). For ES-HPBC patients with smaller tumors (size < 4cm) and fewer LN (LN < 4), the estimated 15-year DSOS rates were: 92% and 79% for high and low ATM group respectively (*p* = 0.0268); and 90% and 74% for low and high Ki67 group respectively (*p* = 0.0004) (Figure 3A and 3B). More importantly, the combination of Ki67 and ATM was a stronger predictor of DSOS than either biomarker alone, as indicated by the higher C-index in the combination (Ki67/ATM: 0.7; Ki67 alone: 0.61; ATM alone: 0.59; *p* < 0.001 for combined Ki67/ATM vs Ki67 alone and *p* = 0.005 for combined Ki67/ATM vs ATM alone) (Figure 2D). Similarly, in the subgroup of ES-HPBC patients defined as tumor size < 4cm and LN < 4, combined biomarkers seem to have a better prognostic performance than either biomarker alone. (Ki67/ATM: 0.68; Ki67 alone: 0.60; ATM alone: 0.59; p = 0.037 for combined Ki67/ATM *vs* Ki67 alone and *p* = 0.053 for combined Ki67/ATM *vs* ATM alone) (Figure 3D). Overall, patients with low Ki67 and high ATM scores showed the most favorable clinical outcome with estimated 15-year DSOS rates of greater than 92% (Figure 2C & 3C).

**Figure 4:**
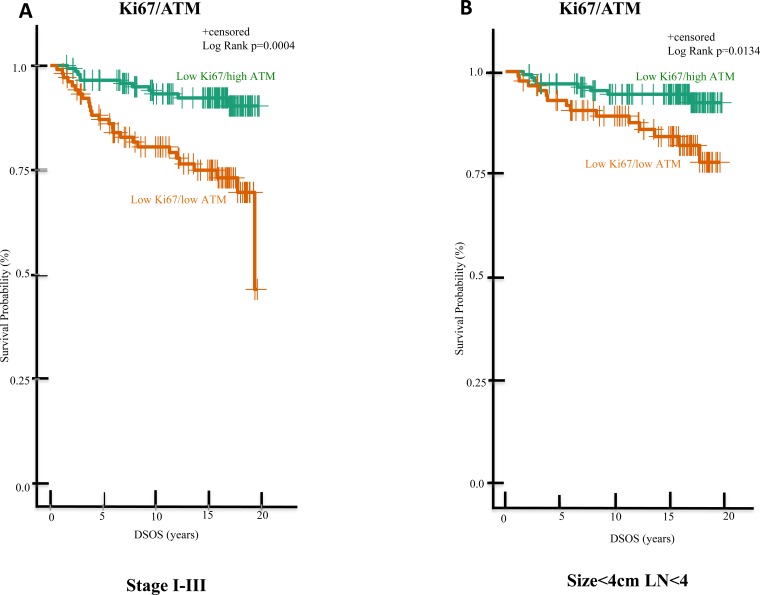

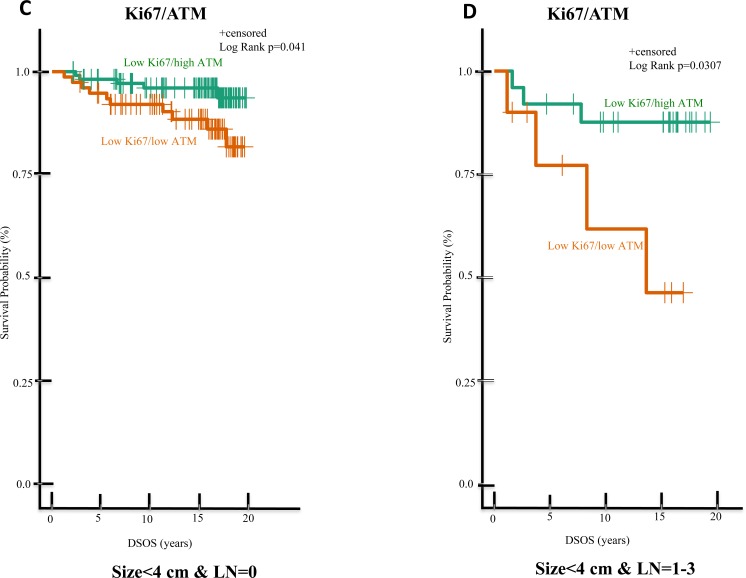
In the low Ki67 subgroup of ES-HPBC, patients with high ATM vs low ATM expression significantly differ in DSOS Shown are Kaplan-Meier analysis of DSOS of ES-HPBC patients with **A.** stage I-III tumors and low Ki67 expression; **B.** low Ki67 expression, smaller tumors (size < 4 cm) and fewer positive nodes (LN < 4), **C.** no positive nodes, low Ki67, and smaller tumors (size < 4 cm), **D.** low Ki67, smaller tumors (size < 4 cm) and LN = 1-3.

We focused the majority of our subsequent statistical analyses on the low Ki67 groups, due to the low numbers of high Ki67 tumors in our final cohort. Low Ki67/low ATM patients had significantly lower 15-year DSOS rates (75%) than the low Ki67/high ATM grou*p* (92%; p = 0.004; Figure 4A). Among patients with smaller tumors (size < 4cm) and fewer positive nodes (LN < 4), the low Ki67/low ATM cases also exhibited poorer DSOS rates (84%) than those with low Ki67/high ATM (94%; *p* = 0.0134; Figure 4B). Similar trends were evident in patients with node-negative patients with smaller tumors (tumor size < 4cm and LN = 0; 96% *vs* 89%; *p* = 0.0411; Figure 4C) and in patients with tumor size < 4cm and LN = 1-3 (88% *vs* 46%; *p* = 0.0307; Figure 4D).

To determine if the combined expression score for Ki67 and ATM was an independent prognostic factor for DSOS in the low Ki67 group, we used Cox proportional hazards regression models. The multivariable model consisted of known clinical prognostic factors such as tumor size, grade, LN status, LVI and age. As expected, tumor size and grade were significantly associated with survival (Table 2). LN status was trending but not significantly associated with survival, likely due to the small sample size of positive LN in this low Ki67 ES-HPBC group. In this subgroup of ES-HPBC with low Ki67 expression, the significant association between combined biomarkers of Ki67/ATM and DSOS persisted in the multivariable model, adjusted for known prognostic variables (*p* = 0.02) (Table 2).

**Table 2:** Low Ki67/high ATM expression independently predicts favorable disease survival in a multivariate model in low Ki67 subgroup of ES-HPBC (Stage I-III)

Variables	pvalue	HR (95%CI)
Low Ki67/high ATM vs Low Ki67/low ATM	0.02	0.36 (0.15-0.88)
Tumor size (T1/2 vs T3/4)	0.01	0.16 (0.04-0.69)
LN status (- vs +)	0.14	0.49 (0.20-1.25)
LVI (_- vs +)	0.16	0.51 (0.20-1.31)
Grade (1/2 vs 3)	<0.0001	0.28 (0.11-0.67)
Age (<65 vs >65)	0.01	0.26 (0.10-0.74)

Stage I-III patients with low Ki67 expression were selected for this analysis. A multivariable model was created using selected known prognostic factors such as tumor size, lymph node (LN) status, lymphovascular invasion (LVI), grade and age. Cox proportional hazards regression methods were used to calculate p values, hazard ratio (HR) and confidence interval (CI) of each variable in the model.

## DISCUSSION

In this study, we showed that the combined Ki67/ATM expression index is a stronger prognostic factor of DSOS than either biomarker alone. When patients were grouped by Ki67 and ATM expression, the ES-HPBC patients with low Ki67 and high ATM scores had the most favorable prognosis. Most importantly, ATM expression identified a subset of patients who had poor prognosis, even with low Ki67 scores. This observation held true across the spectrum of ES-HPBC cases, regardless of stage, tumor size, or node status. To our knowledge, this is the first study to examine the prognostic value of both biomarkers together in breast cancer.

For a variety of reasons, the use of Ki67 for clinical decision-making has been mired in controversy. One such reason surrounds its use as an independent prognostic tool, which is a concern we address in this paper. Several years ago, two extensive meta-analyses were conducted on the use of Ki67 in this context [3, 4]. One was based on 46 studies involving 12,155 patients and the other was based on 43 studies involving 15,790 patients. Both analyses suggested that Ki67 is a useful prognostic tool in both node-positive and node-negative early stage breast cancer. Nevertheless, the independent prognostic value of Ki67 has been frequently challenged [5–8]. In a recent review, Andre and colleagues challenged the use of Ki67 in making clinical decisions for patients with early stage breast cancer and one to three positive axillary nodes [8]. They argue that Ki67 fails to identify a sizable proportion of patients who may have poor prognosis [8]. Our current work highlights the value of secondary biomarkers, such as ATM, in identifying patients whose prognosis is significantly poorer than expected in that low Ki67 subgroup. Fully 35% of patients in our final cohort (*n* = 105/297; Table 1) were categorized as low Ki67/low ATM, and the poorer prognosis of this cohort highlights a significant deficiency in the application of Ki67 alone as a prognostic factor.

One of the common shortcomings with the use of Ki67 as a biomarker is the poor reliability of manual analysis methods. Even with centralized staining, inter-observer concordance with manual scoring is often quite low [10, 22]. For this study, we used an automated digital image analysis platform to score Ki67, which can potentially reduce the effect of human bias (inter- and intra- observer variability) and improve analytical validity. This is an approach that has been recommended by several other studies [11, 12]. The ACIS analysis platform we used in this study offers high concordance with well-trained manual scorers, as documented in similar applications [23–26]. Additionally, utilizing a computer-generated algorithm provides consistent scoring between samples, and minimizes run-to-run variation. One limitation with the Ki67 scoring method in our study is the potential influence from contributing stromal components and tumor-infiltrating intra-epthelial immune cells. Manually selecting tumor regions minimizes potential influence from stromal components; however, it doesn't completely eliminate this potential bias. The addition of a stain for cytokeratin to identify tumor areas may improve the specificity of this biomarker testing, by enhancing definition of tumor areas. Additionally, utilizing multi-staining IHC approaches combined with multispectral imaging that allows for several target antibodies to be used and analyzed on a single slide, biomarkers such as CD68 could also be used to identify, and remove, intratumoral infiltrating leukocytes such as macrophages from Ki67 calculations.

Another source of inconsistency with Ki67 as a prognostic marker in prospective-retrospective clinical studies [3, 4] is the possible qualitative interaction of Ki67 with adjuvant chemotherapy. Qualitative interaction of a biomarker with treatment is defined as patients with a positive biomarker (ie. high Ki67) who are likely to benefit from adjuvant chemotherapy, whereas patients with a negative biomarker (ie. low Ki67) likely derive no benefit or may even be harmed by adjuvant chemotherapy [27]. Therefore, high Ki67 would not necessarily result in a poor prognosis if patients received effective chemotherapy. Similarly, low Ki67 would not necessarily result in a good outcome if patients received harmful chemotherapy. In addition, high Ki67 could predict good or poor outcome depending on if a tumor will respond and be sensitive to a given chemotherapy [28]. In our study, none of patients in the TMA cohort received adjuvant chemotherapy and all patients received tamoxifen as adjuvant endocrine therapy indicating a relatively homogenous study population. Therefore, it is unlikely that the biomarkers investigated in this cohort would succumb to the treatment effect. Biologically, however, the prognostic impact of low Ki67/high ATM is easily justified. A low Ki67 score is indicative of a relatively slow-proliferating, less aggressive tumor; while there were no associations between Ki67/ATM indices and standard clinico-pathological features of the tumors (Table 1), such a correlation would naturally be biased by the various stages at which patients present with disease. As suggested by our previous publication [18], low ATM expression would highlight tumors that are more genetically unstable. The combination of high proliferative rate and low DNA stability is thus clearly a harbinger of relapse, which would, as our data again show, is independently prognostic of DSOS (Table 2 and Appendix 3).

In this study, we identified a significant difference in survival for patients based on Ki67 status. These data contradict those of some large-scale clinical trial studies (BIG 1-98 [29], IBCSGVIII-IX [30], ATAC [31]) which showed that the statistically significant prognostic value of Ki67 was relatively low. For example, the absolute difference of 4-year disease free survival (DFS) in patients with high vs low Ki67 expression was only 3% in the BIG 1-98 trial (93% vs 90%) [29]. In our study, the survival difference was larger between high and low Ki67 expression alone group, which is about 20% difference at 5 years follow up (93% vs 73% respectively, Figure 2B). Although this is likely the effect of sample size and the different treatment regimens in these studies, we believe that this difference is also likely a result from different Ki67 cut-points. Since Ki67 data in our study constitutes a continuous variable generated automatically by the analysis software, we chose to use the X-tile program to define an optimal cut-point. X-tile program is a validated methodology to define biomarker cut-point based on the survival outcome (DSOS) [21]. An additional important observation from our study is that the DSOS difference was more pronounced after five to ten years suggesting that these biomarkers are useful to detect late breast cancer associated mortality in ES-HPBC (Figure 2A-C). We observed a similar trend in DFS using the combined biomarkers in the final cohort as well as the subgroup patients with tumor size <4cm and LN<4 with a significant p value of <0.0001 and 0.0026 respectively (data not shown).

A number of highly reproducible RNA-based multigene analyses have been recently developed and validated for prognostic and predictive purposes of ES-HPBC [13, 32–34]. One of which is Oncotype Dx (Genome Health 21-gene recurrence score), which has been approved in use as a prognostic and predictive biomarker for adjuvant chemotherapy in node negative ES-HPBC [34]. It is notable that proliferation genes, including *Ki67*, are heavily weighted in the Oncotype Dx algorithm to calculate recurrence scores. However, the multigene assays can be logistically difficult and cost-prohibitive for routine clinical use in much of the world. A simplified assay, such as IHC 4 score consisting of standard IHC breast biomarkers such as ER, PR and HER2 as well as Ki67, may have a similar prognostic value when compared to Oncotype Dx in ES-HPBC [31]. Therefore, these observations highlight not only the importance of prognostic value of Ki67 in ES-HPBC but also the fact that when Ki67 is used in the mixture of multigene analysis such as in Oncotype Dx assay and IHC4 score, the prognostic value is significantly enhanced. This supports our finding that the prognostic performance of Ki67 biomarker is significantly improved and refined by ATM status. (C-index in Figure 2D/3D and multivariable analysis in Appendix 3). These two molecular biomarkers represent two hallmarks of cancer—uncontrolled proliferation and genome instability [35]. The combination of these two biomarkers may dictate the distinct luminal tumors’ behavior thereby predicting the prognosis. It would be interesting to further investigate the prognostic value of the combined biomarkers Ki67/ATM when ER/PR intensity scores are taken into consideration in ES-HPBC. The combined IHC biomarkers (Ki67/ATM) with the incorporation of ER/PR in the future could be a much simpler and cheaper tool than RNA based multigene assays if this could be further validated in a larger cohort and prospective clinical studies.

Currently, the predictive validity of Oncotype Dx in ES-HPBC patients with 1-3 LN remains unknown. While breast oncologists are eagerly awaiting the results from three ongoing randomized clinical trials (Rxponder [36], MINDACT [37], OPTIMA [38]) to address this question, Ki67 IHC is intended to guide clinicians to weigh risk-benefit ratio of adjuvant chemotherapy as this is widely available and financially acceptable in most laboratories. Our study revealed that low Ki67 alone may not provide enough prognostic information for clinicians to abort adjuvant chemotherapy in node negative or node positive (LN=1-3) ES-HPBC. In Figures 4A-D, we identified certain high-risk patients with a poorer prognosis if ATM protein expression is low despite a low Ki67 who may warrant further intervention in hopes of reducing their risk of relapse and improving DSOS. This data is consistent with the view presented in a recent review paper cautioning the use of Ki67 in guiding treatment decisions for node positive ES-HPBC [8]. The survival difference between patients with low Ki67/high ATM and low Ki67/low ATM should be, however, interpreted cautiously as our study numbers in these subgroups of node positive (LN-1-3) ES-HPBC are low (n=25 and 10 in low Ki67/high ATM and low Ki67/low ATM groups respectively).

We previously showed that ATM protein expression level in malignant tumors independently predicts DSOS in early stage hormone negative breast cancer. ATM expression was not prognostic in ES-HPBC in a multivariable analysis, however, because ATM protein expression level is strongly associated with known clinico-pathological prognostic factors such as tumor size, grade and LN status in ES-HPBC [18]. In the current study, we confirmed this observation that ATM biomarker alone was not an independent prognostic marker in the multivariable model (p=0.15; Appendix 3); however, the combination of Ki67/ATM biomarkers was an independent prognostic factor (p=0.01; Appendix 3). This further supports our hypothesis that the prognostic performance is significantly strengthened by the combined biomarkers. With regard to ATM staining and analysis, we used fluorescent IHC, cytokeratin masking and automated AQUA image analysis, which can provide greater sensitivity and broader range of protein quantification than the brightfield equivalent. It should be noted, however, that fluorescence IHC and the associated analysis platform are not currently used in clinical practice; therefore some data presented in this study may not be directly applicable. It is however possible that they could be translated into clinically relevant assays upon identification of the levels of protein expression that offer prognostic value.

In spite of the promising nature of our results, there were practical constraints that can only be addressed in a follow-up study. Out of the 532 primary breast tumors in our eligible cohort, only 297 patient samples were suitable for final statistical analysis. Other samples were removed due to inconclusive hormone receptor status, poor tissue quality, or inadequate staining. Consequently, whereas the high Ki67 group showed clearly poorer prognosis than their low Ki67 counterparts (Figure 2B), we were unable to perform extensive sub-group analyses due to the resulting small numbers (n=21 and 26 in ATM high/Ki67 high and ATM low/Ki67 high groups respectively). This may explain the overlap of survival curves for these two subgroups (Figure 2C and Figure 3C and Appendix 2). A larger validation cohort is warranted to see if there is any significant survival difference between patients with ATM high/Ki67 high and ATM low/Ki67 high expression.

In summary, we demonstrated that the combined biomarker analysis (Ki67/ATM) significantly increased the strength of predicting clinical outcome compared to either biomarker (Ki67 or ATM) alone. We identified a subgroup of ES-HPBC patients with low Ki67/high ATM expression who have the most favourable clinical outcome. More importantly, we revealed the significant survival difference in low Ki67 group based on high or low ATM protein expression level. The latter suggests that breast oncologists should not rely on Ki67 expression alone to decide on adjuvant chemotherapy as this may mislead clinicians to undertreat certain patients with ES-HPBC.

## MATERIALS AND METHODS

### Case Selection and Clinical Data Collection

This study was approved by the University of Calgary Conjoint Faculties Research Ethics Board (Ethic ID # REB15-1284), in accordance with the Tri-Council Policy Statement on Research with Human Subjects. Consents directly from patients were not required in this study as per Ethics. The clinical data were collected retrospectively through chart review. The initial clinical cohort consisted of 819 patients diagnosed with breast cancer at the Tom Baker Cancer Centre, as previously described [39]. Within this cohort, 532 patients diagnosed between 1985 and 2000 had resected primary tumor samples available in formalin-fixed paraffin-embedded (FFPE) tissue blocks for construction of a tissue microarray (TMA). Patients who had stage I to III breast cancer, with no evidence of metastatic disease at diagnosis, and had undergone either lumpectomy or mastectomy were included for the final analysis. Estrogen receptor (ER) and progesterone receptor (PR) statuses were assessed by local breast pathologists at the time of diagnosis and later evaluated on TMA using an independent staining method [39]. Samples with discordant results between initial pathological diagnosis and later evaluation on TMA were excluded. HER2 expression was not routinely assessed at the time of diagnosis for this cohort but was tested retrospectively by IHC [39]. Cases found to be HER2 positive were excluded. In the end, 297 cases that were successfully stained with both biomarkers of ATM and Ki67, and with confirmed ER/PR positivity and HER2 negativity were available for statistical analysis (Appendix 1A).

### Tissue Microarray Generation

All archived FFPE blocks were retrieved from Calgary Laboratory Services and reviewed by a pathologist (AMM). TMAs were constructed as previously described [18]. Appropriate controls for the protein targets were available either as tissue controls on the TMA slides or as separate slides within the same staining runs.

### Fluorescence IHC for ATM Detection and Automated Semi-Quantitative Digital Analysis

Fluorescence IHC against ATM was performed on TMA sections as previously described [18]. The following primary antibodies were applied in sequence: rabbit anti-ATM (rabbit monoclonal, clone Y170, ab32420, 1:1000, Abcam, Cambridge, MA, USA), mouse anti-vimentin to identify the stromal compartment (mouse monoclonal, clone V9, 1:5000, DAKO), and guinea pig anti-pan-cytokeratin to identify the epithelial compartment (guinea pig polyclonal, catalog number BP5069, 1:200, Acris Antibodies, San Diego, CA, USA). Automated image acquisition was performed using an Aperio Scanscope® FL (Aperio Inc., Vista, CA, USA) slide scanner. Images were acquired using filters specific for DAPI to define the nuclear compartment, fluorescein isothiocyanate (FITC) to define cytokeratin for the tumor compartment and Cy3 to define ATM. Images were then analyzed using the AQUAnalysis® program, version 2.4.4.1. Briefly, a tumor-specific binary mask was generated to distinguish the cancer cells from surrounding stromal tissue by thresholding the pan-cytokeratin images. All images were processed using optimized threshold values, and manually validated as described [18]. Antibody specificity was validated using the L3 ATM-deficient humansquamous lung carcinoma cell line and the H226 ATM-expressing human squamous cell lung carcinoma cell line as controls in addition to normal tonsil tissue (with no evidence of pathological tonsillitis) treated with either ATM or Ki67 antibody and matched isotype control slides (Figure 1A). ATM expression was calculated as the mean Cy3 pixel intensity within the tumor mask as defined by positive pan-cytokeratin (Figure 1C). For each patient sample, the average AQUA score over triplicate cores was used to define the ATM expression score.

### Brightfield Ki67 Detection and Automated Digital Analysis

Following previously described deparaffinization and rehydration procedures, slides were pre-treated by immersing them in the EnVision FLEX Target Retrieval Solution, low pH (DAKO), and heating to 97°C for 20 minutes in a PT-Link pre-treatment module. Ki67 was detected using the FLEX mouse monoclonal antibody (MIB-1) and EnVision FLEX reagents following the manufacturer's recommended protocol. 3’-diaminobenzidine tetrahydrochloride (DAB) was used to visualize Ki67, while the FLEX hematoxylin counterstain allowed for visualization of the tissue and nuclei. Ki67 was quantified using the ACIS® III Automated Cellular Imaging System (DAKO) [40]. Briefly, TMA slides were scanned and tumor areas were hand selected to exclude stromal areas using the ACIS software. A unique algorithm was developed that identified distinct color thresholds for nuclear hematoxylin and DAB staining, and an overall percent positive score was calculated by taking the total Ki67 (DAB) nuclear score and dividing it by total nuclear score (DAB plus hematoxylin). The final Ki67 score for analysis was obtained by taking an average of up to three tumor cores.

### Statistical analysis

Summary and descriptive statistics were used to describe demographic and clinico-pathological characteristics of patients in the clinical cohort, the TMA cohort and the final cohort for statistical analysis. Histograms were used to evaluate the distribution of Ki67 and ATM expression. In the final cohort, patients were divided into four subgroups based on ATM and Ki67 expression levels. Cut-points to define low and high Ki67 and ATM expression were modeled using X-Tile software [21]. The following comparison was made in the Ki67 low group only. The Student's t-test was used to compare the continuous variable of “age” between low Ki67/high ATM and low Ki67/low ATM groups. Fisher's exact test was used to compare the categorical variables between low Ki67/low ATM and low Ki67/high ATM groups. Disease-free survival (DFS) and breast cancer- or disease-specific overall survival (DSOS) estimates were calculated using the Kaplan-Meier (KM) survival analysis, and log-rank tests were used to compare low and high Ki67/ATM groups. The C-index (concordance index) was used to evaluate the model prediction accuracy [41]. The C-index is defined as the probability of agreement for two randomly selected groups, and the agreement indicates the higher risk group predicted by the model will have shorter survival time. C-index values are ranged from 0 to 1, where 0.5 indicates random prediction, and higher values suggest better model prediction. P values for C-index comparison were obtained by 3000 bootstraps samples. Cox proportional hazards regression methods were used to assess the prognostic effect of Ki67 and ATM combined on DSOS, in a multivariable model with clinical known prognostic factors such as tumor size, grade, LN status, lymphovascular invasion (LVI) and age. The prognostic utility of each biomarker alone and in combination was tested using a backward selection approach to obtain the optimal model. All tests were two-sided, and a p-value of 0.05 was considered statistically significant. All statistical analyses were conducted using R statistical analysis software (Version 3.1.2).

## APPENDIX





## Abbreviations

ATM: ataxia telangiectasia mutated
ES-HPBC: early stage hormone receptor positive breast cancer
TMA: tissue microarray
AQUA: automated quantitative immunofluorescence analysis
FFPE: formalin-fixed paraffin-embedded
ER: estrogen receptor
PR: progesterone receptor
HER2: human membrane epidermal growth factor receptor 2
DSOS: disease specific overall survival
LVI: lymphvascular invasion
LN: axillary lymph node
