# Supplementary material for: Low Ki67/high ATM protein expression in malignant tumors predicts favorable prognosis in a retrospective study of early stage hormone receptor positive breast cancer
Source: Oncotarget. 2016 Oct 12;7(52):85798–812. doi: 10.18632/oncotarget.12622 (PMC5349875; doi:10.18632/oncotarget.12622)
Supplement: Supplementary file 1 [file oncotarget-07-85798-s001.pdf]

Appendix 1A: Consort diagram to demonstrate cohort selection

A

Initial Clinical Cohort (N=819)

↓

Inclusion criteria:
- FFPE blocks of resected primary tumors available

↓

TMA Cohort (N=532)

↓

Inclusion criteria:
- Stage I-III and underwent surgical lumpectomy or mastectomy
- Successful biomarker staining for both ATM and Ki67
- Confirmed ER/PR positivity on TMA spot
- Confirmed HER2 negativity by IHC

↓

Final Cohort (N=297)

Appendix 2: Low Ki67/high ATM expressions in malignant tumor predict favorable DSOS in ES-HPBC (size<4cm & LN=0 or LN=1-3).

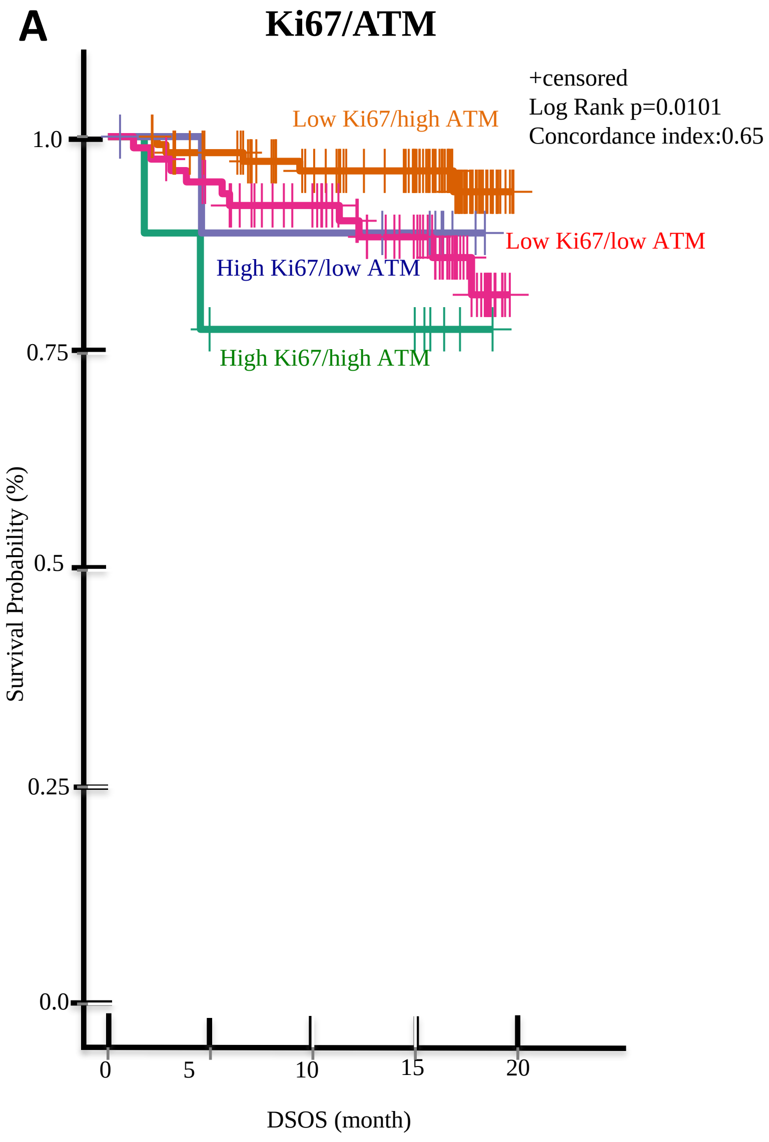

Size<4 & LN=0

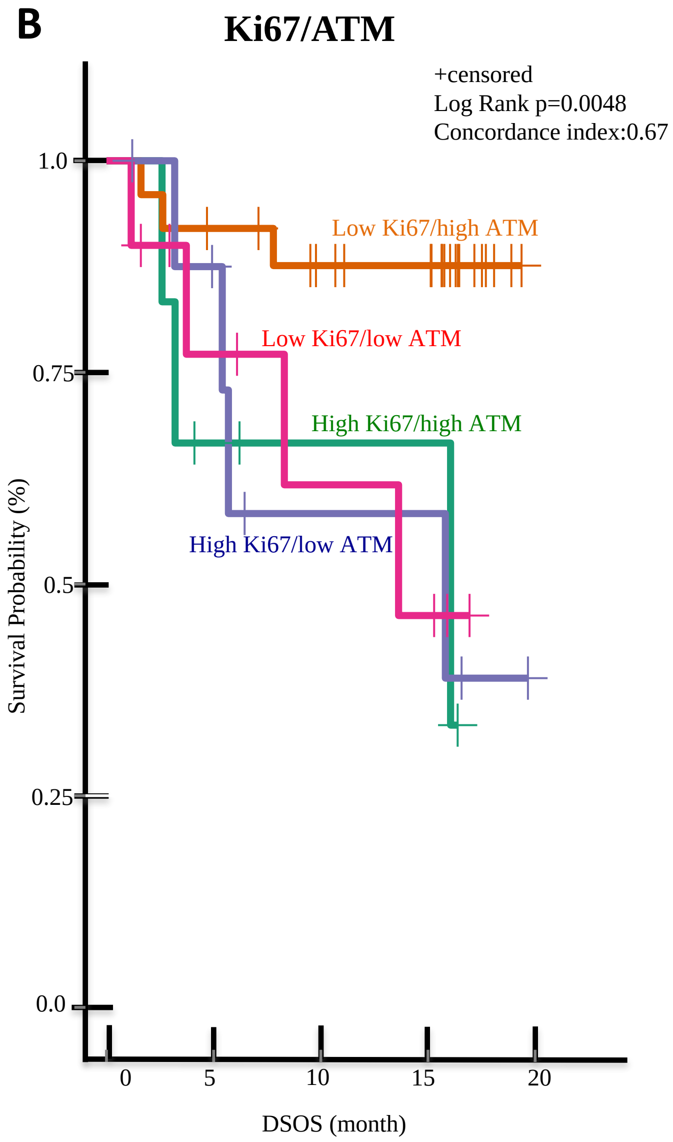

Size<4 & LN=1-3

Appendix 3: Single vs Combined biomarkers predict disease survival in a multivariate model in ES-HPBC (Stage I-III)

A

**Variables**	**p value**	**HR (95%CI)**
Low Ki67/high ATM vs others*	0.01	0.33 (0.15-0.73)
Tumor size (T1/2 vs T3/4)	<0.0001	0.17 (0.05-0.58)
LN status (- vs +)	0.07	0.50 (0.24-1.06)
LVI (_- vs +)	0.26	0.65 (0.31-1.37)
Grade (1/2 vs 3)	0.01	0.37 (0.18-0.76)
Age (<65 vs >65)	0.06	0.51 (0.25-1.04)

* Low Ki67/low ATM, High Ki67/high ATM and High Ki67/low ATM

B

**Variables**	**p value**	**HR (95%CI)**
Low Ki67 vs High Ki67	0.15	0.58 (0.87-1.22)
Tumor size (T1/2 vs T3/4)	<0.0001	0.11 (0.03-0.35)
LN status (- vs +)	0.13	0.56 (0.26-1.19)
LVI (_- vs +)	0.23	0.63 (0.30-1.34)
Grade (1/2 vs 3)	0.01	0.34 (0.16-0.72)
Age (<65 vs >65)	0.06	0.50 (0.25-1.02)

C

**Variables**	**p value**	**HR (95%CI)**
High ATM vs low ATM	0.15	0.61 (0.30-1.20)
Tumor size (T1/2 vs T3/4)	<0.0001	0.14 (0.04-0.49)
LN status (- vs +)	0.07	0.50 (0.24-1.07)
LVI (_- vs +)	0.28	0.66 (0.31-1.40)
Grade (1/2 vs 3)	<0.0001	0.31 (0.15-0.63)
Age (<65 vs >65)	0.07	0.52 (0.26-1.06)
