# Supplementary material for: Low Ki67/high ATM protein expression in malignant tumors predicts favorable prognosis in a retrospective study of early stage hormone receptor positive breast cancer
Source: Oncotarget. 2016 Oct 12;7(52):85798–812. doi: 10.18632/oncotarget.12622 (PMC5349875; doi:10.18632/oncotarget.12622)
Supplement: Supplementary file 2 [file oncotarget-07-85798-s002.doc]

Low Ki67/high ATM protein expression in malignant tumors predicts favorable prognosis in a retrospective study of early stage hormone receptor positive breast cancer

**Appendix 1: Consort diagram to demonstrate cohort selection (A) and Comparison of patients’ characteristics and clinico-pathological features of breast tumors in the initial clinical cohort (N=819), the TMA cohort (N=532) and the final cohort (N=297) (B).**

The initial clinical cohort consisted 819 patients who were diagnosed with invasive breast cancer. Among this initial clinical cohort, FFPE primary tumor samples could be retrieved from 532 patients, which were selected in the TMA cohort. In total, 498 and 348 cases successfully obtained Ki67 and ATM staining respectively. There were 312 cases, which obtained both ATM and Ki67 staining. 15 cases were further excluded from this cohort due to negative or unknown staining of ER and PR on TMA and positive or unknown HER2 results by an independent evaluation. The final 297 cases were met the inclusion criteria for the final statistical analysis. The clinical and histopathological features were summarized and described in numeric numbers and proportions in these three cohorts. Student T-test and fisher exact test were used to compare the continuous variable and categorical variables respectively.

**Appendix 2: Low Ki67/high ATM expressions in malignant tumor predict favorable DSOS in ES-HPBC {size<4cm & LN=0 (A) or size <4cm & LN=1-3(B)}.** Kaplan-Meier analysis of DSOS (**A and B**) of patients in these four groups (low Ki67/high ATM, low Ki67/low ATM, high Ki67/high ATM, high Ki67/low ATM expressions).

**Appendix 3.** **Single (B, C) vs Combined biomarkers (A) predict disease survival in a multivariate model in ES-HPBC (Stage I-III).** Patients in the final cohort (stage I-III) were selected for the analysis. A multivariable model was created using selected known prognostic factors such as tumor size, LN status, LVI, grade and age. Cox proportional hazards regression methods were used to calculate p value, hazard ratio (HR) and confidence interval (CI) of each variable in the model.

**Appendix 1B: Comparison of patients’ characteristics and clinico-pathological features of breast tumors in the initial clinical cohort (N=819), the TMA cohort (N=532) and the final cohort (N=297).**

| **Baseline Characteristics** | **Clinical Cohort**  **N=819** | **TMA Cohort**  **N=532** | **Final Cohort**  **N=297** | **Clinical Cohort vs TMA Cohort vs Final Cohort**  **p value** |
| --- | --- | --- | --- | --- |
| **Age** median (min-max) | 67(32-96) | 66(36-96) | 65(38-96) | 0.519 |
|  |  |  |  |  |
| **Tumor size** |  |  |  |  |
| T1/T2 (<5cm) | 735 (90%) | 500(94%) | 285 (96%) | 0.119 |
| T3/T4 (≥5cm) | 50 (6%) | 21 (4%) | 12 (4%) |  |
| Missing | 34 (4%) | 11 (2%) | 0 (0%) |  |
|  |  |  |  |  |
| **Grade** |  |  |  |  |
| 1 | 199 (24%) | 147 (28%) | 77 (26%) | 0.663 |
| 2 | 382 (47%) | 268 (50%) | 161 (54%) |  |
| 3 | 125 (15%) | 72 (14%) | 47 (16%) |  |
| Missing | 113 (14%) | 45 (8%) | 12 (4%) |  |
|  |  |  |  |  |
| **LVI** |  |  |  |  |
| Negative | 451 (55%) | 314 (59%) | 180 (61%) | 0.831 |
| Positive | 112 (14%) | 79 (15%) | 50 (17%) |  |
| Missing | 256 (31%) | 139 (26%) | 67 (22%) |  |
|  |  |  |  |  |
| **LN** |  |  |  |  |
| 0 | 545 (67%) | 384 (72%) | 220 (74%) | 0.588 |
| >0 | 197 (24%) | 121 (23%) | 77 (26%) |  |
| Missing | 77 (9%) | 27 (5%) | 0 (0%) |  |
|  |  |  |  |  |
| **Stage** |  |  |  |  |
| I | 416 (51%) | 297 (56%) | 168 (57%) | <0.001 |
| II | 242 (30%) | 161 (30%) | 108 (36%) |  |
| III | 69 (8%) | 38 (7%) | 21 (7%) |  |
| IV | 33 (4%) | 10 (2%) | 0 (0%) |  |
| Missing | 59 (7%) | 26 (5%) | 0 (0%) |  |
|  |  |  |  |  |
| **ER/PR status** |  |  |  |  |
| Positive | 484 (59%) | 484 (91%) | 297 (100%) | 0.004 |
| Negative | 13 (2%) | 13 (2%) | 0 (0%) |  |
| Missing | 322 (39%) | 35 (7%) | 0 (0%) |  |
|  |  |  |  |  |
| **HER2 Status** |  |  |  |  |
| Positive | 19 (2%) | 19 (4%) | 0 (0%) | <0.001 |
| Negative | 508 (62%) | 508 (95%) | 297 (100%) |  |
| Missing | 292 (36%) | 5 (1%) | 0 (0%) |  |
|  |  |  |  |  |
| **RT** |  |  |  |  |
| Yes | 492 (60%) | 319 (60%) | 178 (60%) | 0.855 |
| No | 292 (36%) | 199 (37%) | 113 (38%) |  |
| unknown | 35 (4%) | 14 (3%) | 6 (2%) |  |
|  |  |  |  |  |
| **Chemo** |  |  |  |  |
| Anthracycline based | 4 (1%) | 0 (0%) | 0 (0%) | 0.074 |
| Taxanes based | 1 (~0%) | 0 (0%) | 0 (0%) |  |
| CMF or 5FU | 7 (1%) | 0 (0%) | 0 (0%) |  |
| None | 805 (98%) | 532 (100%) | 297 (100%) |  |
| Unknown | 2 (~0%) | 0 (0%) | 0 (0%) |  |
|  |  |  |  |  |
| **Endocrine** |  |  |  |  |
| Tamoxifen | 770 (94%) | 532 (100%) | 297 (100%) | <0.001 |
| AI primary | 150 (18%) | 87 (16%) | 56 (19%) | 0.559 |
